# Lack of Sample Diversity in Research on Adolescent Depression and Social Media Use: A Scoping Review and Meta-Analysis

**DOI:** 10.1177/21677026221114859

**Published:** 2023-02-07

**Authors:** Sakshi Ghai, Luisa Fassi, Faisal Awadh, Amy Orben

**Affiliations:** 1 Department of Psychology, University of Cambridge; 2 MRC Cognition and Brain Sciences Unit, University of Cambridge; 3 Department of Psychiatry, University of Cambridge; 4 School of Clinical Medicine, University of Cambridge

**Keywords:** social media, social-networking sites, depression, sample diversity, adolescence, teen, youths, Global North, Global South

## Abstract

Research on whether social media use relates to adolescent depression is rapidly increasing. However, is it adequately representing the diversity of global adolescent populations? We conducted a preregistered scoping review (research published between 2018 and 2020; 34 articles) to investigate the proportion of studies recruiting samples from the Global North versus Global South and assess whether the association between social media and depression varies depending on the population being studied. Sample diversity was lacking between regions: More than 70% of studies examined Global North populations. The link between social media and depression was positive and significant in the Global North but null and nonsignificant in the Global South. There was also little evidence of diversity within regions in both sampling choices and reporting of participants’ demographics. Given that most adolescents live in the Global South and sample diversity is crucial for the generalizability of research findings, urgent action is needed to address these oversights.

Half of the world’s population is digitally connected (“Bringing Half of the Global Population Online,” n.d.), and mobile-phone ownership is common even for people living in the most deprived regions (World Development Report, 2016). This development has allowed many adolescents to begin accessing social media, fundamentally changing the way they live, communicate, and interact with educational or clinical services (Livingstone et al., 2017). In parallel, there has been a rise in concern among researchers, clinicians, and policymakers that social media use might be driving an increase in adolescent depression or a decline in adolescents’ well-being (Salmela-Aro et al., 2016; Spears et al., 2015; Twenge & Campbell, 2019; Twenge et al., 2017).

However, such links remain complex and heavily debated because studies have mainly been correlational and found mixed results (Odgers & Jensen, 2020) with small effect sizes (Orben & Przybylski, 2019). Thus far, the lack of consensus has mostly been ascribed to individual differences, such as gender, socioeconomic status, and personality traits, influencing the link between social media use and depression or well-being (Odgers & Jensen, 2020). Despite the renewed focus on how these effects may differ from “adolescents to adolescent” (Beyens et al., 2020), little to no attention has been given to how the association of social media and depression might vary in diverse, traditionally underrepresented and understudied communities, for example, populations living in the Global South. Such a lack of sample diversity in psychological science has been broadly recognized (Henrich et al., 2010; Hruschka et al., 2018; Rad et al., 2018). For instance, only 7% of research samples published in psychological science in 2017 examined populations from non-Western countries despite these countries representing the majority of the world population (Rad et al., 2018). Furthermore, clinical trials often recruit samples solely from the Global North and high-resource settings (Hall, 2001; Polo et al., 2019).

The dearth of sample diversity in psychological and clinical research poses a significant challenge to the generalizability of research findings. As Rosmarin (2016) highlighted, “Unless we start to embrace the fact that not all human beings are alike, clinical science will become increasingly irrelevant to most of the population” (p. 702). Note that the cultural contexts in which adolescents live heavily shape their beliefs, and the prevalence of stigma influences perceptions of depressive symptoms in the Global South (Altweck et al., 2015). Furthermore, how social media is accessed, what platforms are used, and how adolescents interact with them vary substantially across contexts and populations around the world (Miller, 2019). Therefore, to make claims about social media use and depression that are relevant to all global populations, researchers need to understand whether (a) there is sufficient sample diversity to generalize existing results and (b) there are any differences in how social media relates to depression between regions.

The issue of sample diversity is twofold. On the one hand, as highlighted above, it concerns the lack of diverse representation across geographic areas (e.g., the Global South). On the other hand, it also relates to the lack of recruiting diverse samples within regions. Research often relies on convenient, unrepresentative, and homogeneous groups (e.g., White, cisgender, educated, middle-class participants), not capturing the diversity present in a certain country or region (Gurven, 2018; Henrich et al., 2010). Moreover, participants’ demographics, such as ethnicity, income, and education, are routinely not measured even in clinical trials, and studies often show high variability in how participants’ demographics are described, making it challenging to gauge sample diversity when reviewing the existing literature (Crosby et al., 2010). For example, a 36-year systematic review examined the extent of sample diversity in 342 randomized control trials of depression and revealed that only fewer than half of the trials fully reported participants’ race/ethnicity (Polo et al., 2019). In fact, Polo et al. (2019) found that most clinical studies rarely reported effects across multiethnic groups, such as Asian American, Native Hawaiian/Pacific Islander, and Native American/Native Alaskan.

Such a lack of sample diversity will directly affect the validity and reliability of research findings and likely lead to more inaccurate generalizations (Hall, 2001). This is especially relevant in research areas with a translational impact on policy and the clinic, for example, the research on the potential links between social media use and adolescent depression, in which results derived from a subgroup of the population might inform policy or clinical decisions that affect other groups traditionally underrepresented in psychological research.

## The Current Study

Given that more than two thirds of the world’s adolescents live in the Global South (Azzopardi et al., 2019), researchers need to investigate the extent to which research quantifying the links between adolescent social media use and depression can be generalized to diverse populations. Furthermore, the field needs to understand whether there are similar issues on a within-regions level such that populations routinely understudied (e.g., disadvantaged groups) in academic research may also be overlooked. To tackle these issues, we hereby examine whether research samples are representative of the world’s adolescent population, whether the association between depression and social media use differs depending on the global population being studied, and whether research samples sufficiently represent within-regions diversity. Overall, we aim to address generalizability and representativeness issues in this heavily expanding research area.

To understand differences in the global populations studied, we use the label “Global North and Global South” as a metacategory to classify economically developed and developing countries (Haug et al., 2021). For example, the Global North corresponds to much of the Western, developed world, including, for example, the United States, United Kingdom, Europe, Australia, Japan, Canada, and New Zealand, whereas the Global South relates to the low- and middle-income countries in Asia, Africa, Latin America, and the Caribbean. The Global North–South terminology is now increasingly used in academic scholarship across the social and behavioral sciences to highlight the geopolitical disparities across regions (Dados & Connell, 2012) and examine the domination of Western scholarship in research (Castro Torres & Alburez-Gutierrez, 2022).

We conducted a preregistered scoping review examining whether studies quantifying the link between adolescent social media use and depression, published in the last 3 years, relied on skewed or diverse samples at two levels: between and within regions. First, to understand the between-regions diversity, we analyzed the extent to which studies relied only on samples from the Global North and then tested whether the metacorrelation between depression and social media use differed for studies sampling populations from the Global North versus the Global South. Second, to gauge the extent of within-regions diversity, we considered whether studies tested samples that included disadvantaged groups and were representative of the general population and investigated whether studies reported participants’ demographics beyond age and gender (e.g., education, income, and ethnicity).

## Method

Because research findings on the impact of social media use across diverse populations are limited, we adopted a scoping review methodology (Arksey & O’Malley, 2007). Compared with systematic reviews, scoping reviews do not aim to provide a critically appraised and synthesized answer to a particular question. Instead, they aim to identify knowledge gaps in the literature and offer an overview of existing evidence. Scoping reviews have been increasingly used in medicine (Agarwal et al., 2019; Barclay & Hilton, 2019), clinical psychiatry (Jacobsen et al., 2018; Leijten et al., 2021; Reinders et al., 2019), and, most recently, psychology (Premachandra & Lewis, 2022). Similar to systematic reviews, scoping reviews use rigorous and transparent ways to analyze the evidence base on a topic (Munn et al., 2018). For instance, they include exhaustive searches for resources and extract data in a reproducible and structured way. Our preregistered scoping review was executed using a five-stage methodological framework outlined by Arksey and O’Malley (2007): (a) identifying the research question, (b) identifying relevant studies, (c) study selection, (d) charting the data, and (e) collating, summarizing, and reporting the results. In addition, we followed the guidelines proposed by the Preferred Reporting Items for Systematic Reviews and Meta-Analyses extension for scoping-reviews checklist (Tricco et al., 2018). Finally, all analyses were performed with the R statistical programming language (Version 4.1.2; packages used in the analysis listed in our OSF project, https://osf.io/nv9p3/).

### Identifying the research question

The present review addresses whether (a) the current research into social media’s relation with depression has sufficiently diverse samples between global regions (Global North vs. Global South), (b) the link between social media use and depression differs according to global region, (c) the samples collected are representative and/or understudied (e.g., disadvantaged groups), and (d) there is adequate reporting of participants’ demographics to gauge other forms of diversity.

### Identifying relevant studies

Our search terms, which we applied using a title and abstract search, included items targeting social media, depression, and adolescent populations combined using the “AND” operator (for details on the search string, see Tables D and E in the Supplemental Material available online). We included both general social media search terms (OR: “social media,” “online social networking,” “internet use,” “social-media,” “online-community,” “online-communities,” “social-app,” “social-apps,” “social-networking-app,” “social-networking-apps,” “social-networking-site,” “social-networking-sites”) and the 15 most popular social media websites worldwide (Statista, 2021; OR: “Facebook,” “YouTube,” “WhatsApp,” “Messenger,” “WeChat,” “Instagram,” “QQ,” “Tumblr,” “Qzone,” “Tik-Tok,” “Sina-Weibo,” “Twitter,” “Reddit,” “Baidu-Tieba,” “LinkedIn”). We also included search terms related to depression (OR: “depression,” “depressive disorder,” “depression,” “depressed,” “depressive”) and adolescence (OR: “adolescent,” “child,” “adolescent development,” “adolescent,” “adolescence,” “teen,” “teens,” “teenager,” “teenagers,” “youth,” “youths,” “young,” “young-adult,” “young-adults,” “student,” “students,” “undergraduate,” “postgraduate”).

### Inclusion criteria

To identify relevant studies, we applied a variety of search criteria. First, we searched for articles published in the last 3 years (2018–2020), a time window that allowed us to capture (a) the exponential growth in social media research and (b) the time when social media use in the Global South had passed a critical threshold (Poushter et al., 2018). We therefore narrowed our search to 2018 to 2020 to appropriately reflect the narrowing digital gaps between the Global North and the Global South and increase our chances of finding published research from around the world. Second, we searched for studies of adolescents, defined as individuals between the ages of 10 and 24 (Sawyer et al., 2018). Third, we used three databases for our literature search: PubMed, PsycInfo, and Global Health. These spanned different academic fields so we could prioritize both medical and psychological research. Articles were selected by screening the title and abstract; however, the article was read at full length when a decision could not be made on the basis of this information.

**Fig. 1.**
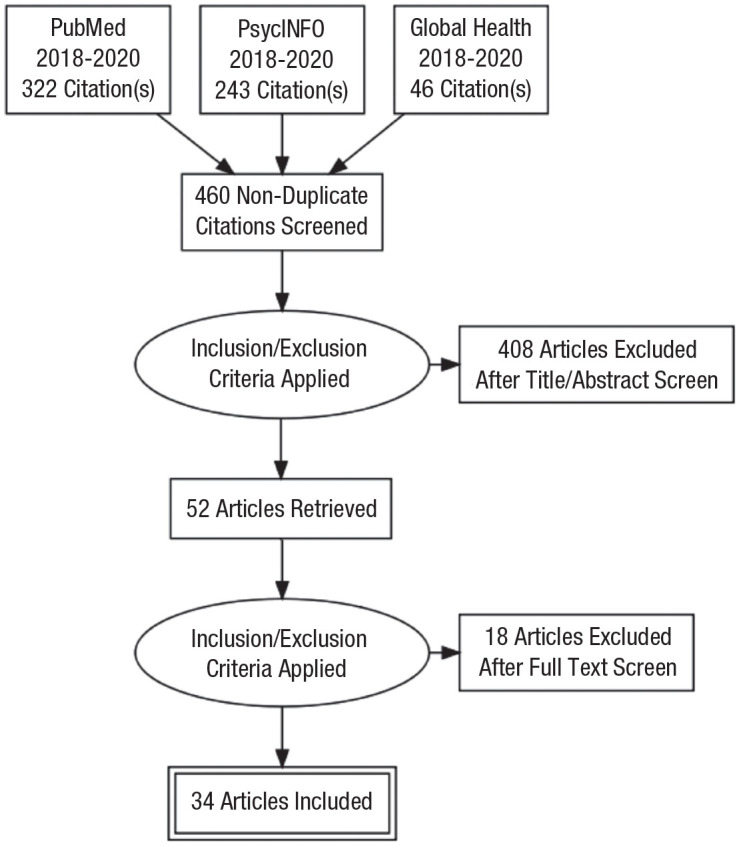
Flowchart of the study selection process. The chart depicts the number of identified, screened, and included articles in the present scoping review.

The initial search found 460 nonduplicate items (Fig. 1), which we then reviewed, applying different inclusion criteria. First, only peer-reviewed research was included. Second, we considered only articles using empirical or mixed-methodological approaches (with quantitative analyses focusing on the relationship between social media use and depression) published from 2018 to 2020. Dissertations, non-peer-review articles, and articles that did not include empirical work (e.g., systematic reviews, literature reviews, meta-analyses, metasynthesis, scoping reviews, narrative reviews, rapid reviews, or qualitative approaches) were excluded. Third, we included only articles that used at least one quantitative measure of each variable: depression, social media use, and the relationship between social media use and depression. Finally, we included all languages in our search; most retrieved studies were written in English except for one study published in Persian.

### Study selection

Initially, F. Awadh retrieved 611 articles (across all three databases) and exported them to Rayyan (Ouzzani et al., 2016). At this stage, duplicates were identified and removed, leaving 460 nonduplicate citations to be screened. Then, F. Awadh and A. Orben screened the studies’ titles/abstracts according to the inclusion/exclusion criteria. As a result, 408 articles were excluded; 52 articles were retrieved, of which three were marked as “maybe include”; and 21 conflicts were identified (often those studies with abstracts that poorly described the study measurements). To resolve this, articles were full-text screened and discussed in depth until both F. Awadh and A. Orben came to a unanimous agreement. As a result, 18 articles were further excluded, bringing the final inclusion count to 34 articles (Fig. 1).

### Charting the data

Using a prespecified coding spreadsheet, L. Fassi coded all articles, and S. Ghai coded a random selection of 25% of articles. Out of the articles coded twice, more than 95% of agreement was reached. The coding spreadsheet included the title, year, journal, authors, study design (i.e., cross-sectional or longitudinal), and geographical areas (i.e., whether the samples were collected in the Global North vs. Global South and the country). To code the geographical areas, we employed the regional classifications provided by the United Nation’s specialized agency, the official source for information and technology statistics (International Telecommunications Union, 2021). If the geographical location of the studied sample was not mentioned, we used the primary author affiliation as a proxy. Furthermore, the coding spreadsheet included the depression and social media use measures used in each study (open response: type of measure and name of the employed questionnaire) and the correlation reported between these measurements (for more details about variations in measurement, see Tables A and B in the Supplemental Material).

We also coded whether the study collected a convenient, representative, and/or disadvantaged sample (dichotomous; yes/no) and whether the study reported participants’ demographics, such as age, gender, education, ethnicity, and income (dichotomous; reported/not reported). A study was coded as using a representative sample if the authors directly mentioned that the study was representative or that they used probability sampling. On the contrary, we coded a study as using a convenient sample if the authors tested student populations or otherwise collected participant data via nonprobability sampling. Furthermore, we used a dichotomous measure of disadvantage in which we coded a study as including a disadvantaged population (1) if the study mentioned any type of disadvantage. Specifically, we included in this category studies that mentioned economic disadvantage (i.e., income or unemployment), educational disadvantage, family disadvantage (i.e., family not finished school), health disadvantage (i.e., mental or physical illness), or environmental disadvantage (i.e., dirty surroundings or neighborhoods) in their sample characteristics (Estes, 2014).

Finally, we also coded whether the study mentioned any caveat associated with the sample they recruited (i.e., small sample size, lack of ethnic diversity, limitations to generalizability across populations) and noted the citation counts of each article (for more details, see Fig. C in the Supplemental Material). We also provide details in the Supplemental Material on why we deviated from our original plan of coding these studies along the WEIRD acronym to determine whether the study samples were Western, Educated, Industrialized, Rich, and Democratic (Henrich et al., 2010).

### Meta-analysis

We ran all analyses in the R statistical programming language (Version 4.1.2); the full list of packages used for the analysis can be found in the open-access code shared on the OSF (https://osf.io/nv9p3/). We conducted an exploratory meta-analysis to test whether the correlation between social media use and depression differed in studies that tested samples from the Global North versus the Global South. The association between the two variables was defined as positive when increased social media use was associated with an increase in depressive symptoms. For all analyses, we employed an a priori statistical significance level of α = .05.

Among the selected studies, some reported an effect size other than a correlation coefficient (e.g., a regression coefficient) to describe the association between social media use and depression. Hence, we first included studies that reported a correlation coefficient in the article’s main text or supplementary materials (*N* = 22). If a study reported an effect size other than a correlation, the authors were contacted by email and asked to provide a correlation coefficient (e.g., Pearson’s *r* or Spearman’s *r*). L. Fassi and S. Ghai contacted 12 authors, of which six replied and provided the coefficient(s) of interest. The remaining six studies were excluded from the meta-analysis. The data file available on OSF provides detailed information regarding which authors were contacted (https://osf.io/nv9p3/).

Raw effect sizes were transformed into normalized correlation coefficients (Fisher’s *z*) to stabilize the variances. For effect sizes initially reported as Spearman’s *r*s, we first transformed them to *r* and then transformed them to Fisher’s *z*. We used the following equation to perform this transformation, *r* = 2sin(*r*s(π/6)), as reported by Rupinski and Dunlap (2016). All correlations included in the meta-analysis were transformed from Fisher’s *z* back to Pearson’s *r* for reporting.

#### Metacorrelation model

A random-effects model was applied to calculate overall summary effect sizes. To interpret the outcomes of the correlational meta-analyses, in line with Cohen (1988), we took correlation coefficients of 0.10 to be small effect sizes, 0.30 to be medium effect sizes, and 0.50 or greater to be large effect sizes. Note that some studies contributed to a larger extent to the meta-analysis because they reported multiple effect sizes. Thus, to account for variance inflation emerging from dependent observations for different measures for the same participants, we used cluster-robust variance estimation (RVE) based on the sandwich method with adjusted estimators for small samples and correlated effects weighting scheme. A default value of *r* = .80 was specified in the RVE (Fisher & Tipton, 2015; Hedges et al., 2010). To examine the variance and heterogeneity among the considered effect sizes, we computed *Q*, *T*^2^, and *I*^2^, interpreting statistically significant *Q* values to indicate heterogeneity and *I*^2^ values of approximately 25%, 50%, and 75% to indicate low, moderate, and high heterogeneity, respectively.

#### Moderator analysis

We applied a moderator analysis to examine the contribution of the global area (Global North vs. Global South) as a source of heterogeneity in the observed correlations. This variable satisfied the minimum requirement of four effect sizes per moderator level, which was defined on the basis of published literature (Fu et al., 2011; Parry et al., 2021; Saiphoo & Vahedi, 2019). Given our interest in disentangling how the association between social media use and depression may differ between the Global North and the Global South, for this moderator, in addition to metaregression models, we estimated separate random-effects models to produce summary effect sizes for each subgroup.

## Results

In this study, we investigated whether a lack of sample diversity skews research on the association between adolescent depression and social media use. To that end, we examined (a) whether the Global South is adequately represented in the existing literature; (b) how the link between depression and social media differs depending on whether the Global North or Global South is studied; (c) whether there is sufficient sample diversity within regions, as indicated by the use of disadvantaged and nonconvenient samples; and (d) whether the reporting of participants’ demographics was enough to assess sample diversity.

### Sample diversity between regions

**Fig. 2.**
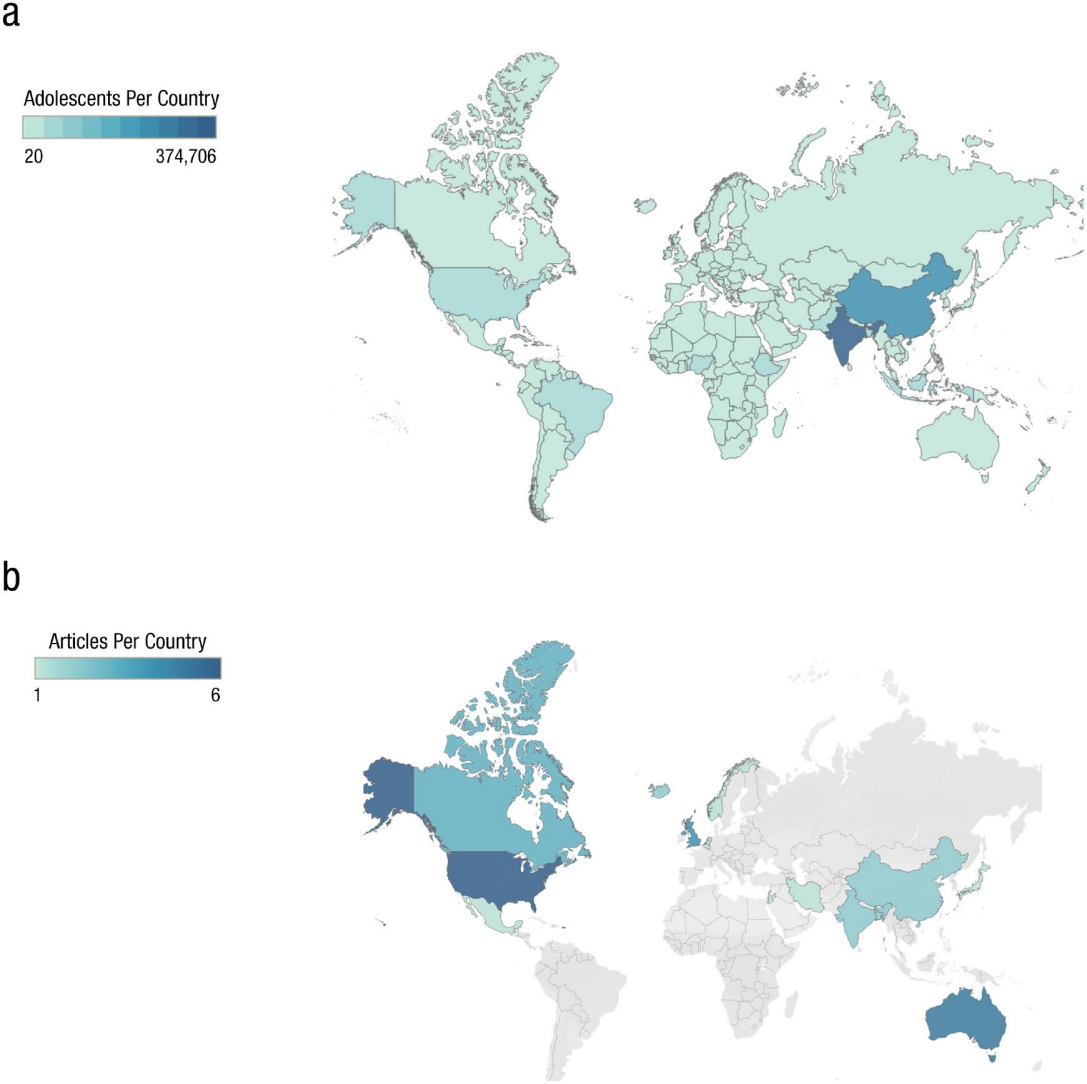
World map showing the size of each country’s adolescent population in relation to the percentage of studies found in our scoping review. (a) This figure represents in thousands the adolescent population (ages 10–24) living in each global region, according to data retrieved from the United Nations (Department of Economic and Social Affairs, Population Division, 2020). The darker blue areas represent more densely populated regions, and the lighter blue areas represent less populated regions (for more details regarding each country’s adolescent population, refer to Fig. B and Table C in the Supplemental Material available online). (b) This figure represents a breakdown of the geographical region of each study included in our scoping review. Specifically, the majority of studies tested Global North samples: 26% from North America, 26% from Europe, and 15% from Oceania. On the contrary, only 21% of the studies tested an Asian sample (including Japan), 9% from the Middle East, a small fraction of the studies from Latin America (3%), and not a single study from Africa. These maps were created using Tableau Public (©2022 MapBox **©**Open Street Maps).

Out of the 34 considered studies, more than 70% (24 studies) examined participants from the Global North, and fewer than 30% (10 studies) examined a population from the Global South (Fig. 2b). In the Global North, a wide range of countries was represented: Canada, the United States, the United Kingdom, Belgium, Netherlands, Norway, Iceland, Japan, and Australia. Most studies were from North America (26%; nine studies), followed closely by Europe (26%; nine studies) and Oceania (15%; five studies). In the Global South, we found only 3% (one study) from Latin America (Mexico), 9% (three studies) from the Middle East (Jordan/Lebanon/Iran), 18% (six studies) from Asia (China/Bangladesh/India), and no studies representing Africa. Although Japan falls under Asia, it was coded as Global North because it is a high-income country, as specified by the International Telecommunications Union (2021), and accounts for 3% of the total sample. Hence, the overreliance on Global North samples suggests that the majority of adolescent populations around the world are not well represented in this research area.

**Fig. 3.**
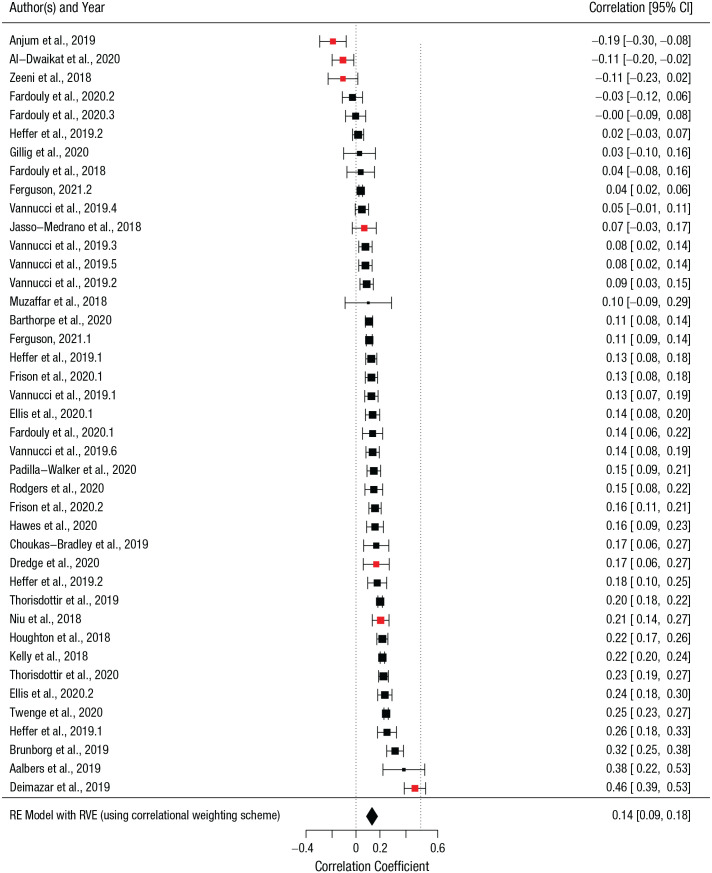
Forest plot of effect sizes on the association between social media use and depression. Individual Pearson’s *r* coefficients are depicted as filled squares with the size indicating the relative weight of each effect size estimate in the meta-analysis. The color indicates the global area: red squares for samples from the Global South and black squares for samples from the Global North. The filled black diamond represents the overall summary effect size across all studies (*r* = .14, 95% confidence interval [CI] = [.09, .20], *p* < .001), calculated using robust variance estimation to account for dependencies between effect sizes coming from the same study. The error bars and diamond width represent the 95% CIs for the effect sizes. The dashed reference line at the intercept for *r* = .5 represents the point from which the magnitude of the association would be sufficient to conclude that the association between social media use and depression is moderate.

To examine whether the link between depression and social media differed depending on whether the population being studied was from the Global North or South, we ran a metacorrelation analysis including 40 effect sizes from 28 studies (Figure 3). Across these comparisons, the total sample size was 83,066. On average, a comparison involved 2,077 participants (*SD* = 3,612, *Mdn* = 909, minimum = 102, maximum = 16,398). Our meta-analysis, investigating whether the association of social media use and adolescents depression varied between the Global North and Global South, found a positive yet small correlation on average across all samples. This result aligned with previous literature (Berryman et al., 2018; Orben, 2020; Valkenburg et al., 2022) showing that increased social media use is associated with small increases in depression (or decreases in well-being). Note that because the reviewed articles were all based on correlational evidence, no causal claims can be made regarding the directionality of the link between social media use and depression.

We found a high degree of heterogeneity in the considered correlation coefficients, *Q*(39) = 738.87, *p* < .001, indicating that the examined studies did not share a common effect size, ranging both from positive to negative and from strong to weak associations. The RVE analysis further confirmed this: *T*^2^ = 0.014 (*SE* = 0.003), *I*^2^ = 96.31% (for a more detailed explanation of publication bias, refer to Fig. A and Meta-Analysis Specifications in the Supplemental Material). To identify potential outliers, we conducted both outlier and influence diagnostics (extrapolating Cook’s distance, covariance ratios, and diagonal elements of the hat matrix) with the *metafor* package (Viechtbauer, 2010). Influence diagnostics (ID) indicated one outlier (ID = 11, area = Global South, *r* = .462, 95% confidence interval [CI] = [.39, .53], residuals = 3.30). A sensitivity analysis excluding this outlier produced a summary effect size for the Global South similar to the original analysis (*r* = .01, 95% CI = [−.17, .18], *p* = .910). Therefore, this study was included in the follow-up analysis.

To examine whether the link between depression and social media differed according to global area, we calculated metacorrelation coefficients separately for effect sizes from the Global South (seven effect sizes from seven studies) and the Global North (33 effect sizes from 21 studies). We found that the pooled effect size for the Global North was significant yet small (*r* = .16, 95% CI = [.13, .20], *p* < .001). On the contrary, the pooled effect size for the Global South was close to zero and nonsignificant (*r* = .08, 95% CI = [−.14, .29], *p* = .426). Both effect sizes were associated with high heterogeneity: *I*^2^ = 95.99 for the Global North, and *I*^2^ = 95.78 and Global South.

Given our interest in examining the role of global areas as a potential source of heterogeneity and given the availability of sufficient data (Global South: *k* = 7; Global North: *k* = 33), we also ran a moderator analysis. We found no evidence in support of a moderating role of Global South versus Global North on the association between adolescent depression and social media use (β = −0.08, *SE* = 0.09, 95% CI = [−0.29, 0.13], *p* = .411), and heterogeneity remained high: *I*^2^ = 95.94%. Therefore, we cannot currently conclude that the global area is a significant source of heterogeneity in the association between social media use and depression. When we ran a sensitivity analysis removing the previously identified outlier (ID = 11, area = Global South, *r* = .462, 95% CI = [.39, .53], residuals = 3.30), we still found a nonsignificant (although stronger) contribution of the global area as a moderator of the relationship between depression and social media use (β = −0.15, *SE* = 0.07, 95% CI = [−0.32, 0.01], *p* = .068).

Note that despite following standard procedures, the statistical power of the moderator analyses was limited by the number of available studies. Given that only a small and heterogeneous number of effect sizes (7/40 effect sizes) was reported for the Global South, CIs capturing the metacoefficient for this level of the moderator were very large.

### Sample diversity within regions

**Fig. 4.**
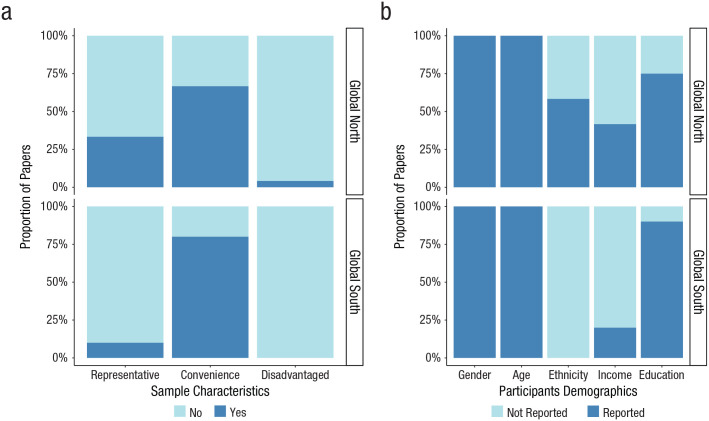
Bar graph displaying the proportion of articles in relation to the recruited samples and the reported participants’ demographics. The bar graphs show differences in the extent to which (a) studies from the Global North compared with the Global South recruited samples that were representative, convenient, and disadvantaged and (b) studies from the Global North compared with the Global South reported participants’ demographics (gender, age, ethnicity, income, and education).

To examine the extent to which research findings are generalizable within countries, we investigated (a) the sampling characteristics of the recruited participants and (b) whether studies adequately reported their demographics (Fig. 4).

To ensure diverse representation across research samples, we specifically focused on the use of convenient, representative, and disadvantaged samples (Fig. 4a). Our results showed that most studies used convenience samples across the two regions: 80% of samples from the Global South were convenient, compared with 62% from the Global North. A limited number of studies tested representative populations (e.g., using probability sampling): 33% of samples from the Global North compared with 10% from the Global South. Finally, 4% of samples from the Global North were disadvantaged compared with 0% from the Global South.

We also examined whether studies provided sufficient detail to judge sample diversity within regions (Fig. 4b). We found that information about gender and age was reported by all studies irrespective of geographical area. Education was reported by most studies: 90% of studies from the Global South reported information about adolescents’ current educational level compared with 75% of studies from the Global North. On the contrary, information on income and ethnicity was not always reported. Forty-two percent of studies from the Global North reported information about income. This percentage was even lower for studies from the Global South (20%). Furthermore, 58% of studies from the Global North mentioned information about participants’ ethnicity compared with 0% of studies from the Global South. In conclusion, studies in the Global North and Global South did not comprehensively report the sociodemographic diversity of adolescents.

### Discussion

The evident lack of diverse samples in clinical-psychological research has been widely documented (Coakley et al., 2012). In the current study, we draw attention to the way this issue may bias an area of research that is currently undergoing exponential growth and is closely related to clinical and policy applications around the world: the study of social media use and adolescent depression.

Our findings show that (a) a striking 70% of reviewed studies recruited samples from the Global North, whereas only 30% of studies recruited samples from the Global South; (b) the metacorrelation between depression and social media use was small, positive, and significant in samples from the Global North, whereas it was null and nonsignificant in samples from the Global South; (c) studies from both the Global North and Global South heavily relied on convenient and nonrepresentative samples, with little research being carried out on disadvantaged adolescent populations; and (d) although age and gender were always reported and education was often reported, ethnicity and income were missing in the majority of studies.

Consistent with previous reviews examining the extensive lack of sample diversity in psychological research (Arnett, 2016; Rad et al., 2018), we found an overall lack of sample diversity across regions. It is remarkable that such little research comes from the Global South even though this area is home to the majority of the world’s adolescents and social media users (Statista, 2021; UNICEF Data, 2019). Hence, the lack of sample diversity in existing research can strongly bias the understanding of how social media use is associated with adolescents’ depression. This is especially the case given that attitudes toward depression and technology use will likely vary widely between the Global North and South (Altweck et al., 2015; Livingstone et al., 2019). Our meta-analysis, investigating whether the association of social media use and adolescents’ depression varied between the Global North and Global South, found—on average across all samples—a positive yet small correlation, a result aligned to previous literature (Orben, 2020). Among the factors that may explain such variability (e.g., gender-specific effects or methodological choices), our focus was on sample diversity, an often neglected yet crucial source of heterogeneous outcomes in clinical psychology. We found a positive and significant correlation between social media and depression in the Global North; in contrast, in the Global South, the correlation was null and nonsignificant. We ran moderation analysis to further explore the role of global area as a potential source of heterogeneity among the examined effect sizes. In this case, our results showed that global area (Global North vs. Global South) was not a significant moderator of the metacorrelation coefficient.

Together, these findings provide mixed evidence regarding whether differences exist in the association of depression and social media use between the Global North and the Global South. However, we want to highlight that the discrepancies emerging from these results might stem from the limited and highly heterogeneous correlation coefficients extracted from studies based in the Global South. Specifically, only seven out of 40 effect sizes were from this global area. Moreover, the metacorrelation coefficient for the Global South presented a broad confidence interval, spanning from a lower bound of –.14 to an upper bound of .29. These considerations, in turn, highlight the existing lack of literature on globally diverse samples. Therefore, more studies with samples from the Global South will be essential in allowing researchers to reach adequately powered conclusions about potential differences in the association between social media use and adolescent depression worldwide.

To further examine whether research samples provided a representative snapshot of adolescent populations, we considered the sample characteristics of each study. We found that the majority of studies relied on convenience sampling with almost negligible amounts of disadvantaged samples. These results were in a similar direction in both geographical regions such that almost no studies focused on disadvantaged samples in the Global South. In line with previous studies on participants’ demographic diversity (Hughes et al., 2016), we also found that although gender and age were always reported and education was often reported, ethnicity and income were rarely reported in the reviewed studies. Therefore, there was not only little variance between regions, but there was also insufficient information on whether the samples were socioeconomically or ethnically diverse within those regions. Furthermore, future research may also need to account for other aspects of participants’ demographics, such as religion, sexual orientation, or urban and rural differences.

We underscore four important caveats to our study. First, we conducted our search on three databases—Global Health, PsycInfo, and Pubmed, which are primarily written in English. Because we did not include any region-specific (e.g., Africa or Asia) databases, we could have inadvertently missed research published in multilingual journals. Nevertheless, we ensured that no study was excluded because of its language, and after applying our inclusion criteria, our final sample included a study published in Persian. Future research should consider looking into multilingual open-access databases such as the Directory of Open Access Journals, which represents research published in 130 countries across 80 languages. Second, despite conducting a systematic search, we restricted our time frame to 3 years. Hence, we could have excluded key articles that study the relationship between social media use and depression in underrepresented samples. Third, scoping reviews do not typically review the quality of evidence (Davis et al., 2009). Thus, we might have overlooked variability in, for example, the quality of measurements used to describe social media use and depression. Finally, all the considered studies relied on correlations to capture the relationship between depression and social media use. Hence, no causal inferences can be drawn regarding whether increased social media use leads to depression or vice versa.

## Conclusion

In our scoping review and meta-analysis, two thirds of reviewed studies sampled populations from the Global North. Because the majority of the world’s adolescent population lives in the Global South, this bias can have clinical repercussions and needs to become an urgent area of focus. However, scientists also need to expand research in the Global North to ensure that samples are not homogeneous and include socially disadvantaged populations.

Sample diversity is indeed crucial to developing a nuanced perspective on adolescent depression and its relation to social media use. To account for both between- and within-regions variations, future research should first and foremost diversify its sampling methodologies and broaden the reporting of participants’ demographics. However, to truly create a more enduring change in the field, the field needs to (a) cocreate research and collaborate with diverse researchers globally to advance the scholarship on social media use and depression and (b) urgently prioritize and fund more work that advances underrepresented research samples living in both the Global North and Global South. Only by doing this will researchers account for sample diversity as a source of heterogeneity in research outcomes and prioritize underrepresented samples to ultimately reduce global disparities in knowledge production.

## Supplemental Material

sj-docx-1-cpx-10.1177_21677026221114859 – Supplemental material for Lack of Sample Diversity in Research on Adolescent Depression and Social Media Use: A Scoping Review and Meta-Analysis Click here for additional data file. Supplemental material, sj-docx-1-cpx-10.1177_21677026221114859 for Lack of Sample Diversity in Research on Adolescent Depression and Social Media Use: A Scoping Review and Meta-Analysis by Sakshi Ghai, Luisa Fassi, Faisal Awadh and Amy Orben in Clinical Psychological Science

## Transparency

*Action Editor:* Pim Cuijpers

*Editor:* Jennifer L. Tackett

*Author Contribution(s)*

Contributorship was documented with cRediT (Holcombe et al., 2020):

**Sakshi Ghai:** Conceptualization; Data curation; Formal analysis; Methodology; Project administration; Visualization; Writing – original draft; Writing – review & editing; Joint first authorship.

**Luisa Fassi:** Data curation; Formal analysis; Visualization; Writing – original draft; Writing – review & editing; Joint first authorship.

**Faisal Awadh:** Conceptualization; Methodology; Writing – original draft.

**Amy Orben:** Conceptualization; Methodology; Project administration; Supervision; Writing – original draft; Writing – review & editing.

**Open Practices:** This article has received the badges for Open Data, Open Materials, and Preregistration. More information about the Open Practices badges can be found at http://www.psychologicalscience.org/publications/badges.
