# Supplementary material for: Lack of Sample Diversity in Research on Adolescent Depression and Social Media Use: A Scoping Review and Meta-Analysis
Source: Clin Psychol Sci. 2023 Feb 7;11(5):759–72. doi: 10.1177/21677026221114859 (PMC10491482; doi:10.1177/21677026221114859)
Supplement: sj-docx-1-cpx-10.1177_21677026221114859 – Supplemental material for Lack of Sample Diversity in Research on Adolescent Depression and Social Media Use: A Scoping Review and Meta-Analysis [file sj-docx-1-cpx-10.1177_21677026221114859.docx]

- **Supplementary Materials**

**Deviations from the Original Pre-registration**

***WEIRD Coding***

Our initial plan was to code for the WEIRD acronym (Henrich et al., 2010) (i.e., determine whether the samples of the study were white, educated, industrialized, rich and democratic using international indices). Our work on a different project after the pre-registration but prior to this study’s completion taught us that such a process is not helpful, and it is unfeasible to code some of these characteristics (Ghai, 2021). Further, there are “poor, rural, and uneducated subpopulations living in countries glossed with the ‘WEIRD’ label” (Gurven, 2018) and, therefore, such coding would not help us gain insights into the regional and national diversity of our samples. We, therefore, decided to focus this study on coding the geographical area of Global North and Global South.

***Student Sample***

We had planned to code for whether a sample was collected from a student sample. However, during the process of study coding, we realized that since our focus was on adolescent samples, such coding was redundant and would not help us gain better insights into sample demographics. We, therefore, restricted our coding to only convenient samples that can include but are not restricted to student samples.

**Social Media and Depression Measures**

We examined the measures employed by the reviewed studies to capture social media use and depression. Studies quantified social media use in three ways: a validated questionnaire, a free response, or a Likert scale response (Supplementary Table A). Most studies (56%) measured time spent on social media through an item associated with a Likert scale. For instance, participants were asked to report on how many hours per day, on average, they spent using social media platforms choosing between different options (e.g., less than 10 min, 10–30 min, 31–60 min, 1–2 hr, 2–3 hr, 3–5 hr, 5–10 hr, to more than 10 hr). On the contrary, 16% of the studies asked participants to fill in their answers as a free-response. In comparison, 28% of studies used an existing and validated questionnaire (e.g., Social Network Usage Questionnaire). Overall, most studies focused on measuring time spent on social media, while five studies examined a specific social media platform (such as Facebook or Qzone).

All the studies included in this scoping review employed an existing questionnaire to capture adolescents’ depression, except for one study that used a single-item question. As summarized in Supplementary Table B, the Center for Epidemiological Studies Depression Scale (CES-DC) was the most widely used questionnaire, followed by the Mood and Feelings Questionnaire (MFQ) and the Patient Health Questionnaire (PHQ).

**Supplementary Table A.**

*Summary of the social media questionnaires used in the reviewed studies and their characteristics*

| Questionnaire name | Acronym | Frequency | Items | Range |
| --- | --- | --- | --- | --- |
| The Technology Use Questionnaire | TUQ | 1 | 20 | 0-8 |
| Passive and Active Social Media Use | P/ASMU | 1 | 7 | 0-100 |
| Social Network Usage Questionnaire | SNUQ | 1 | 19 | 0-5 |
| Bergen Facebook Addiction Scale | BFAS | 1 | 6 | 1-5 |
| Psycho‐Social Aspects of Facebook Use | PSAFU | 1 | 9 | 1-5 |
| Screen Based Media Use Scale | SBMUS | 1 | / | / |
| Cuestionario de Adiccction on a Redes Sociales | ARS | 1 | 24 | 1-5 |
| Multidimensional Scale of Facebook Use | MSF | 1 | 7 | 1-8 |
| Media and Technology Usage and Attitudes Scale | MTUAS | 1 | 15 | 1-10 |

**Supplementary Table B.**

*Summary of the depression questionnaires used in the reviewed studies and their characteristics*

| Questionnaire name | Acronym | Frequency | Items | Range |
| --- | --- | --- | --- | --- |
| Center for Epidemiological Studies Depression Scale (for children, adolescents, or in short form) | CES-DC | 9 | 9-20 | 0/4/1-4/1-5/0-3 |
| Mood and Feelings Questionnaire (short form or standard version) | SMFQ | 6 | 13 | 0-2/1-5 |
| Patient Health Questionnaire | PHQ | 4 | 9 | 0-4 |
| Brief Symptom Inventory | BSI | 2 | 6 | 1-5 |
| Depression Anxiety Stress Scale | DASS-21 | 2 | 21 | 0-3 |
| Original Symptom Checklist | OSC | 2 | 10 | 1-4 |
| Beck's Depression Inventory | BDI | 1 | 21 | / |
| Children’s Depression Inventory 2 | CDI-2 | 1 | 12 | 1-3 |
| The Clinically Useful Depression Outcome Scale | / | 1 | 4 | 1-4 |
| Anhedonic depression subscale of the Mini-MASQ | MASQ | 1 | 8 | 1-5 |
| Spence Children’s Anxiety Scale | SCAS | 1 | / | 1-4 |

**Meta-analysis Specifications**

***Inclusion of Longitudinal Studies***

For longitudinal studies that reported correlation coefficients at multiple time points, we only selected coefficients at the first time point, unless the contacted authors provided us with coefficients from a different time point or a pooled coefficient (if this was the case, we specified it in the data file).

***Publication Bias***

Egger’s regression test, incorporating RVE per the Egger–sandwich test (Viechtbauer, 2010), indicated no evidence of small study bias in this sample (*β* = -1.21, *SE*= 1.35 *p*=0.80). In line with this result, visual inspection of the contour-enhanced funnel plot (Supplementary Figure A) does not indicate asymmetry. In addition, the absence of evident over-representation of effect sizes in the highlighted significance contours suggests no evidence of publication bias.

**Supplementary Figure A.**


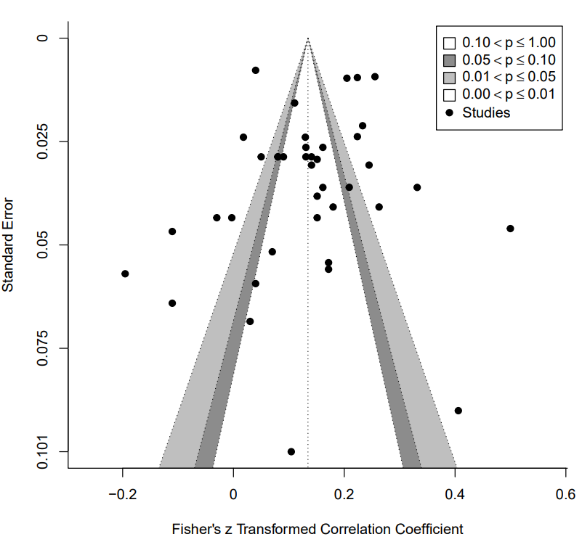
*Contour-enhanced funnel plot depicting observed effect sizes versus their standard errors for comparisons concerning social media use and depression*

*Note.* The vertical line indicates the estimated effect sizes. The shaded bands represent the significant contours presented in the legend.

**Sample Limitations**

To further examine whether samples within the Global North and South reflected on the sample limitations associated with their sampling strategy, we considered whether studies from the Global North and the Global South differ in the extent to which the authors’ self-reflected on study limitations (Supplementary Figure B). We coded whether the study mentioned any caveat associated with the sample they tested (1; e.g., small sample size, lack of ethnic diversity, limitations to generalizability across populations), while we coded the study as 0 if it did not mention any caveat.

We found that both regions acknowledged the lack of diversity in their samples equally. For example, a study from the US that we reviewed noted that “The current sample was homogenous, consisting of young women from a single college in the southeastern United States who predominantly (67%) were white. The pattern of results remained the same when controlling ethnicity, but further work will be needed to understand racial differences in young women’s experiences of appearance-related social media consciousness (ASMC). In addition, results may not generalize to other young female social media users, and the findings from this preliminary examination of ASMC will need to be replicated in larger, more heterogeneous samples of women and adolescent girls (Choukas-Bradley et al., 2019.” While authors who conducted a study in China reported that “A final limitation of the current study was that data was only collected from one school in one province of China and that this school was representative of mostly upper‐class students with well-educated parents. Such sample characteristics are not necessarily representative of all Chinese adolescents (Dredge, & Chen, 2020).”

**Supplementary Figure B.**

*World map showing the size of each country’s adolescent population in relation to the percentage of studies found in our scoping review (descending order)*

**
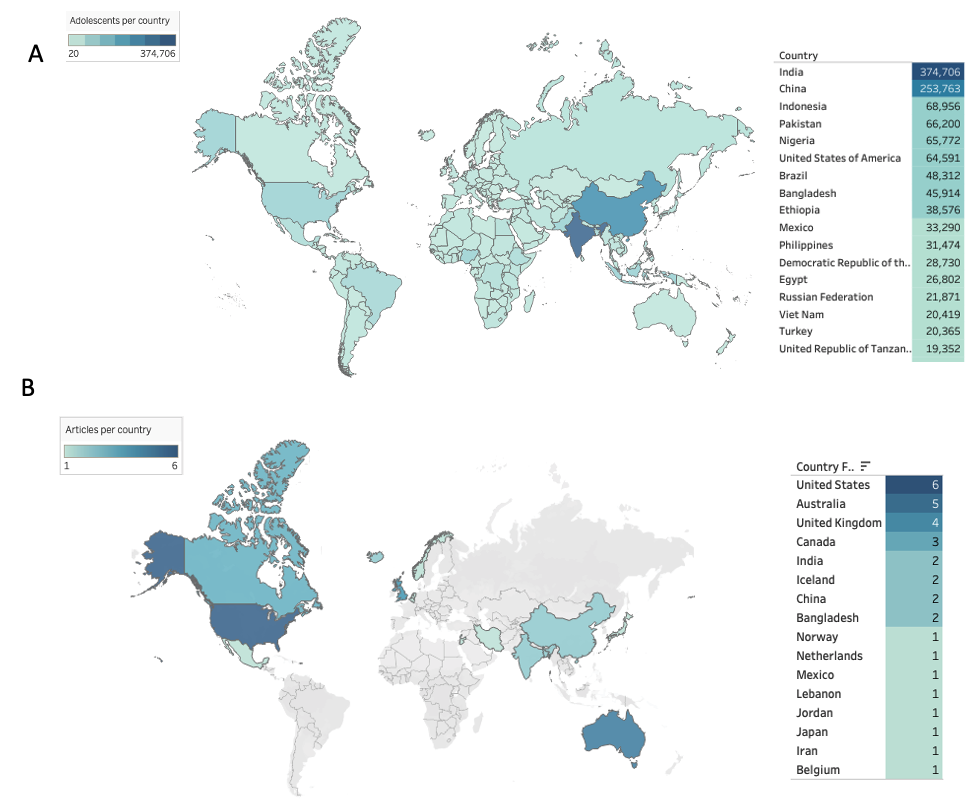
**

*Note.* These maps were created using Tableau (**©2022** MapBox **©**Open Street Maps). In Figure B, we do not provide the complete list of countries and the respective adolescent populations that are reported in Supplementary Table C. Further, the adolescent population data for Greenland was missing from this database, and therefore, we excluded Greenland when creating this figure.

**Supplementary Table C.**

*The size of each country’s adolescent population (United Nations Department of Economic and Social Affairs, Population Division, 2020 data)*

| Countries | Adolescent Population 2020  *(Thousands)* |
| --- | --- |
| Afghanistan | 13748.04 |
| Albania | 590.134 |
| Algeria | 9731.584 |
| Angola | 10764.35 |
| Antigua and Barbuda | 21.446 |
| Argentina | 10614.05 |
| Armenia | 545.427 |
| Aruba | 21.575 |
| Australia | 4725.048 |
| Austria | 1384.969 |
| Azerbaijan | 2054.585 |
| Bahamas | 97.291 |
| Bahrain | 273.539 |
| Bangladesh | 45913.65 |
| Barbados | 55.281 |
| Belarus | 1354.355 |
| Belgium | 1969.953 |
| Belize | 117.191 |
| Benin | 3895.322 |
| Bhutan | 210.301 |
| Bolivia | 3362.885 |
| Bosnia and Herzegovina | 562.443 |
| Botswana | 675.773 |
| Brazil | 48312.5 |
| Brunei Darussalam | 100.284 |
| Bulgaria | 985.348 |
| Burkina Faso | 6949.437 |
| Burundi | 3768.062 |
| Cabo Verde | 145.896 |
| Cambodia | 4628.375 |
| Cameroon | 8596.457 |
| Canada | 6334.789 |
| Central African Republic | 1747.379 |
| Chad | 5574.229 |
| Channel Islands | 28.217 |
| Chile | 3896.788 |
| China | 253762.8 |
| China, Hong Kong SAR | 927.005 |
| China, Macao SAR | 82.859 |
| China, Taiwan Province of China | 3691.09 |
| Colombia | 12541.34 |
| Comoros | 270.848 |
| Congo | 1734.231 |
| Costa Rica | 1119.068 |
| Côte d'Ivoire | 8643.339 |
| Croatia | 644.237 |
| Cuba | 1948 |
| Curaçao | 31.075 |
| Cyprus | 235.119 |
| Czechia | 1532.728 |
| Dem. People's Republic of Korea | 5502.102 |
| Democratic Republic of the Congo | 28729.73 |
| Denmark | 1049.894 |
| Djibouti | 269.528 |
| Dominican Republic | 2875.438 |
| Ecuador | 4682.519 |
| Egypt | 26802.03 |
| El Salvador | 1798.907 |
| Equatorial Guinea | 403.489 |
| Eritrea | 1145.872 |
| Estonia | 195.496 |
| Eswatini | 389.835 |
| Ethiopia | 38575.85 |
| Fiji | 230.018 |
| Finland | 919.913 |
| France | 11580.5 |
| French Guiana | 82.882 |
| French Polynesia | 65.592 |
| Gabon | 602.177 |
| Gambia | 785.04 |
| Georgia | 704.47 |
| Germany | 12484.05 |
| Ghana | 9477.057 |
| Greece | 1598.271 |
| Grenada | 24.836 |
| Guadeloupe | 86.673 |
| Guam | 40.988 |
| Guatemala | 5674.1 |
| Guinea | 4504.479 |
| Guinea-Bissau | 631.02 |
| Guyana | 222.746 |
| Haiti | 3411.727 |
| Honduras | 3079.369 |
| Hungary | 1487.15 |
| Iceland | 66.947 |
| India | 374706.3 |
| Indonesia | 68956.28 |
| Iran (Islamic Republic of) | 17304.16 |
| Iraq | 12510.63 |
| Ireland | 958.431 |
| Israel | 2029.036 |
| Italy | 8675.954 |
| Jamaica | 727.77 |
| Japan | 17264.05 |
| Jordan | 3120.004 |
| Kazakhstan | 3820.969 |
| Kenya | 17983.47 |
| Kiribati | 34.529 |
| Kuwait | 740.32 |
| Kyrgyzstan | 1615.09 |
| Lao People's Democratic Republic | 2158.25 |
| Latvia | 258.255 |
| Lebanon | 1728.169 |
| Lesotho | 632.744 |
| Liberia | 1638.097 |
| Libya | 1727.925 |
| Lithuania | 376.486 |
| Luxembourg | 105.736 |
| Madagascar | 9018.672 |
| Malawi | 6576.77 |
| Malaysia | 7978.826 |
| Maldives | 110.491 |
| Mali | 6785.435 |
| Malta | 65.977 |
| Martinique | 71.517 |
| Mauritania | 1423.682 |
| Mauritius | 269.275 |
| Mayotte | 89.448 |
| Mexico | 33290.28 |
| Micronesia (Fed. States of) | 34.947 |
| Mongolia | 728.942 |
| Montenegro | 119.805 |
| Morocco | 8971.54 |
| Mozambique | 10508.02 |
| Myanmar | 14675.66 |
| Namibia | 770.745 |
| Nepal | 9341.654 |
| Netherlands | 2974.439 |
| New Caledonia | 66.552 |
| New Zealand | 949.43 |
| Nicaragua | 1824.871 |
| Niger | 7987.987 |
| Nigeria | 65771.76 |
| North Macedonia | 363.745 |
| Norway | 996.3 |
| Oman | 859.361 |
| Pakistan | 66199.71 |
| Panama | 1075.672 |
| Papua New Guinea | 2754.692 |
| Paraguay | 2014.066 |
| Peru | 7742.697 |
| Philippines | 31474.03 |
| Poland | 5675.119 |
| Portugal | 1555 |
| Puerto Rico | 538.149 |
| Qatar | 465.843 |
| Republic of Korea | 7969.502 |
| Republic of Moldova | 660.404 |
| Réunion | 202.704 |
| Romania | 3085.183 |
| Russian Federation | 21870.73 |
| Rwanda | 4089.356 |
| Saint Lucia | 40.205 |
| Saint Vincent and the Grenadines | 26.718 |
| Samoa | 58.57 |
| Sao Tome and Principe | 72.786 |
| Saudi Arabia | 7193.412 |
| Senegal | 5382.744 |
| Serbia | 1528.9 |
| Seychelles | 20.229 |
| Sierra Leone | 2621.217 |
| Singapore | 903.331 |
| Slovakia | 836.2 |
| Slovenia | 292.692 |
| Solomon Islands | 209.82 |
| Somalia | 5471.865 |
| South Africa | 15333.27 |
| South Sudan | 3644.066 |
| Spain | 6991.986 |
| Sri Lanka | 4881.871 |
| State of Palestine | 1608.135 |
| Sudan | 14283.05 |
| Suriname | 150.967 |
| Sweden | 1670.913 |
| Switzerland | 1319.053 |
| Syrian Arab Republic | 4928.625 |
| Tajikistan | 2622.17 |
| Thailand | 13300.22 |
| Timor-Leste | 436.501 |
| Togo | 2658.729 |
| Tonga | 33.22 |
| Trinidad and Tobago | 271.506 |
| Tunisia | 2461.225 |
| Turkey | 20365.17 |
| Turkmenistan | 1470.28 |
| Uganda | 15777.61 |
| Ukraine | 6529.564 |
| United Arab Emirates | 1558.744 |
| United Kingdom | 11717.11 |
| United Republic of Tanzania | 19351.6 |
| United States of America | 64591.14 |
| United States Virgin Islands | 20.502 |
| Uruguay | 732.994 |
| Uzbekistan | 8243.19 |
| Vanuatu | 92.835 |
| Venezuela (Bolivarian Republic of) | 7260.196 |
| Viet Nam | 20419.25 |
| Western Sahara | 148.534 |
| Yemen | 9675.52 |
| Zambia | 6297.12 |
| Zimbabwe | 4937.784 |

**Supplementary Figure C.**

*Bar graph displaying the proportion of authors that discussed limitations of their samples*


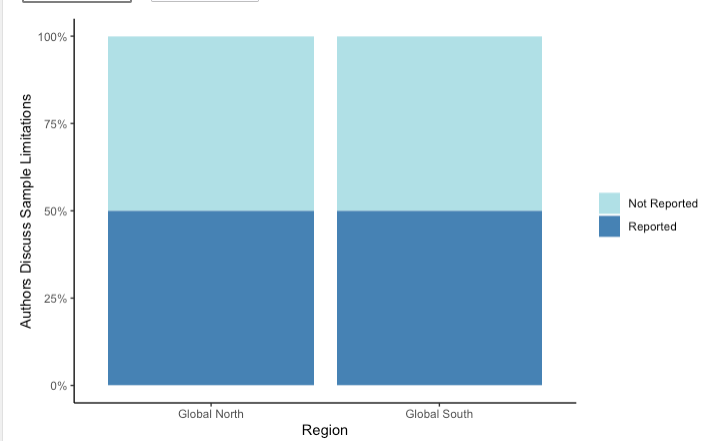


**Citation Counts**

As citation counts are an important indicator of measuring the impact of an author or a study, we coded for the number of citations of each paper to compare whether they would differ by geographical area. The citation count for each study was retrieved using the citation reference on the Web of Science (Web of Science, 2020). We did not find a statistically significant difference between the citation counts in the Global North (*M*=25.9, *SD*=25.2) and the Global South group (*M*=15.5, *SD*=16.7), *t(25)*=1, *p*=0.2.

**Supplementary Table D.**

*Search Strategy Used for the Scoping Review on Pubmed*

|  | **Search String – 20^th^ January 2021** | **No.** |
| --- | --- | --- |
|  | Accessed via Cambridge Medical Library (<https://libguides.cam.ac.uk/az.php>) and selected **Pubmed** database. |  |
| **1** | Click ‘Advanced Search’  · ("social media"[Mesh] OR "online social networking"[Mesh] OR "internet use"[Mesh] or "social-media"[Title/Abstract] OR "online-community"[Title/Abstract] OR "online-communities"[Title/Abstract] OR "social-app"[Title/Abstract] OR "social-apps"[Title/Abstract] OR "social-networking-app"[Title/Abstract] OR "social-networking-apps"[Title/Abstract] OR "social-networking-site"[Title/Abstract] OR "social-networking-sites"[Title/Abstract] OR "Facebook"[Title/Abstract] OR "YouTube"[Title/Abstract] OR "WhatsApp"[Title/Abstract] OR "Messenger"[Title/Abstract] OR "WeChat"[Title/Abstract] OR "Instagram"[Title/Abstract] OR "QQ"[Title/Abstract] OR "Tumblr"[Title/Abstract] OR "Qzone"[Title/Abstract] OR "Tik-Tok"[Title/Abstract] OR "Sina-Weibo"[Title/Abstract] OR "Twitter"[Title/Abstract] OR "Reddit"[Title/Abstract] OR "Baidu-Tieba"[Title/Abstract] OR "LinkedIn"[Title/Abstract])  AND    · ("adolescent"[Mesh] OR "child"[Mesh] OR "adolescent development"[Mesh] OR "adolescent"[Title/Abstract] OR "adolescence"[Title/Abstract] OR "teen"[Title/Abstract] OR "teens"[Title/Abstract] OR "teenager"[Title/Abstract] OR "teenagers"[Title/Abstract] OR "youth"[Title/Abstract] OR "youths"[Title/Abstract] OR "young"[Title/Abstract] OR "young-adult"[Title/Abstract] OR "young-adults"[Title/Abstract] OR "student"[Title/Abstract] OR "students"[Title/Abstract] OR "undergraduate"[Title/Abstract] OR "postgraduate"[Title/Abstract])  AND    · ("depression"[Mesh] OR "depressive disorder"[Mesh] OR "depression"[Title/Abstract] OR "depressed"[Title/Abstract] OR "depressive"[Title/Abstract]) | **545** |
| **2** | · Apply Publication Year **2018-2020** | **322** |

**Supplementary Table D.**

*Search Strategy Used for the Scoping Review on PsycInfo*

|  | **Search String – 20^th^ January, 2021** | **No.** |
| --- | --- | --- |
|  | Accessed via EbscoHost (**PsycInfo**) (<https://libguides.cam.ac.uk/az.php>) and searched for PsycInfo database. |  |
|  | · Selected’ Boolean/Phrase’ under section ‘search modes’  · Select Apply equivalent subjects  · Select **Advanced Search** |  |
| 1 | Select relevant Search Field as detailed below:    1. (TI, Title) DE social media or DE online social networks or DE internet usage or social-media or online-community or online-communities or social-app or social-apps or social-networking-app or social-networking-apps or social-networking-site or social-networking-sites or Facebook or YouTube or WhatsApp or Messenger or WeChat or Instagram or QQ or Tumblr or Qzone or Tik-Tok or Sina-Weibo or Twitter or Reddit or Baidu-Tieba or LinkedIn  **OR**  2. (AB, Abstract) DE social media or DE online social networks or DE internet usage or social-media or online-community or online-communities or social-app or social-apps or social-networking-app or social-networking-apps or social-networking-site or social-networking-sites or Facebook or YouTube or WhatsApp or Messenger or WeChat or Instagram or QQ or Tumblr or Qzone or Tik-Tok or Sina-Weibo or Twitter or Reddit or Baidu-Tieba or LinkedIn | **30,068** |
| 2 | Select relevant Search Field as detailed below:    1. (TI, Title) DE adolescent or DE child or DE adolescent development or adolescent or adolescence or teen or teens or teenager or teenagers or youth or youths or young or young-adult or young-adults or student or students or undergraduate or postgraduate  **OR**  2. (AB, Abstract) DE adolescent or DE child or DE adolescent development or adolescent or adolescence or teen or teens or teenager or teenagers or youth or youths or young or young-adult or young-adults or student or students or undergraduate or postgraduate | **1,202,681** |
| 3 | Select relevant Search Field as detailed below:    1. (TI, Title) DE depression or DE depressive disorders or depression or depressed or depressive  **OR**  2. (AB, Abstract) DE depression or DE depressive disorders or depression or depressed or depressive | **312,118** |
| 4 | · Select above three searches in ‘Search History’ and click ‘Search with AND’. | **558** |
| 5 | · Apply Publication Year **2018-2020** | **243** |

**Supplementary Table E.**

*Search Strategy Used for the Scoping Review on Global Health*

|  | **Search String – 20^th^ January, 2021** | **No.** |
| --- | --- | --- |
|  | Accessed via EbscoHost (**Global Health**) (<https://libguides.cam.ac.uk/az.php>) and searched for Global Health database. |  |
|  | · Selected’ Boolean/Phrase’ under section ‘search modes’  · Select Apply equivalent subjects  · Select **Advanced Search** |  |
| 1 | Select relevant Search Field as detailed below:    1. (TI, Title) DE social media or DE online social networks or DE internet usage or social-media or online-community or online-communities or social-app or social-apps or social-networking-app or social-networking-apps or social-networking-site or social-networking-sites or Facebook or YouTube or WhatsApp or Messenger or WeChat or Instagram or QQ or Tumblr or Qzone or Tik-Tok or Sina-Weibo or Twitter or Reddit or Baidu-Tieba or LinkedIn  **OR**  2. (AB, Abstract) DE social media or DE online social networks or DE internet usage or social-media or online-community or online-communities or social-app or social-apps or social-networking-app or social-networking-apps or social-networking-site or social-networking-sites or Facebook or YouTube or WhatsApp or Messenger or WeChat or Instagram or QQ or Tumblr or Qzone or Tik-Tok or Sina-Weibo or Twitter or Reddit or Baidu-Tieba or LinkedIn | **7.778** |
| 2 | Select relevant Search Field as detailed below:    1. (TI, Title) DE adolescent or DE child or DE adolescent development or adolescent or adolescence or teen or teens or teenager or teenagers or youth or youths or young or young-adult or young-adults or student or students or undergraduate or postgraduate  **OR**  2. (AB, Abstract) DE adolescent or DE child or DE adolescent development or adolescent or adolescence or teen or teens or teenager or teenagers or youth or youths or young or young-adult or young-adults or student or students or undergraduate or postgraduate | **195,518** |
| 3 | Select relevant Search Field as detailed below:    1. (TI, Title) DE depression or DE depressive disorders or depression or depressed or depressive  **OR**  2. (AB, Abstract) DE depression or DE depressive disorders or depression or depressed or depressive | **46,504** |
| 4 | · Select above three searches in ‘Search History’ and click ‘Search with AND’. | **83** |
| 5 | · Apply Publication Year **2018-2020** | **46** |

**References**

Choukas-Bradley, Sophia, Jacqueline Nesi, Laura Widman, & M. K. Higgins. (2019). Camera-Ready: Young Women’s Appearance-Related Social Media Consciousness.” *Psychology of Popular Media Culture* 8, no. 4: 473–81. <https://doi.org/10.1037/ppm0000196>

Dredge, R, & Chen, S. (2020). Chinese Online Gamers versus Nongamers: A Difference in Social Media Use and Associated Well‐being and Relational Outcomes? *Psychology in the Schools*. <https://doi.org/10.1002/pits.22418>

Ghai, S. (2021). It’s time to reimagine sample diversity and retire the WEIRD dichotomy. *Nature*  *Human Behaviour*, *5*(8), 971–972. <https://doi.org/10.1038/s41562-021-01175-9>

Gurven, M. D. (2018). Broadening horizons: Sample diversity and socioecological theory are essential to the future of psychological science. *Proceedings of the National Academy of Sciences*, *115*(45), 11420–11427. <https://doi.org/10.1073/pnas.1720433115>

Henrich, J., Heine, S. J., & Norenzayan, A. (2010). The weirdest people in the world? *Behavioral and Brain Sciences*, *33*(2–3), 61–83. <https://doi.org/10.1017/S0140525X0999152X>

Viechtbauer, W. (2010). Conducting Meta-Analyses in *R* with the metafor Package. *Journal of Statistical Software*, *36*(3). <https://doi.org/10.18637/jss.v036.i03>

*Web of Science* (2020, May). <https://access.clarivate.com/login?app=wos&alternative=true&shibShireURL=https:%2F%2Fwww.webofknowledge.com%2F%3Fauth%3DShibboleth&shibReturnURL=https:%2F%2Fwww.webofknowledge.com%2F%3Fmode%3DNextgen%26action%3Dtransfer%26path%3D%252Fwos%252Fwoscc%252Fbasic-search%26DestApp%3DUA&referrer=mode%3DNextgen%26path%3D%252Fwos%252Fwoscc%252Fbasic-search%26DestApp%3DUA%26action%3Dtransfer&roaming=true>
